# Women physicians and the COVID-19 pandemic: gender-based impacts and potential interventions

**DOI:** 10.1080/07853890.2022.2164046

**Published:** 2023-01-03

**Authors:** Padmini D. Ranasinghe, Ashley Zhou

**Affiliations:** School of Medicine, Johns Hopkins University, Baltimore, MD, USA

**Keywords:** Women physicians, burnout, COVID-19, gender equity

## Abstract

**Aim: **These are extraordinary times caused by the first global pandemic in our modern era. Physicians and other frontline healthcare providers face unique challenges, for which they have had little formal preparation. This combination of challenge and deficit leads to significant negative impacts, not only on what medical practices and health care systems can deliver to the public, but also on the individual healthcare providers themselves.

**Methods:** In this essay, we specifically address women physicians, and explore the considerable impact they bear from the COVID-19 pandemic, particularly in the contexts of response to stress, social isolation, work-life integration, and autonomy. Because the language we use is important, we think it necessary to clarify that when we refer to ‘women physicians,’ we are referring to physicians that self-identify as women, and we acknowledge that not all the references we cite may use the same definition.

**Results: **We offer several potential interventions that turn the challenges women physicians are facing into opportunities to address longstanding inequity. These interventions include tackling barriers to work-life balance, addressing gender and maternal bias, and promoting women physician representation in leadership.

**Conclusion: **The COVID-19 pandemic is likely to become a chronic part of our lives; protecting vulnerable populations, such as women physicians, through thoughtful intervention is paramount.

**KEY MESSAGES**
- Women physicians experience considerable adversity during the COVID-19 pandemic, particularly in the contexts of response to stress, social isolation, work-life integration, and autonomy.
- These challenges create opportunities for interventions to improve equity in medicine during the COVID-19 pandemic and in the long-term, including tackling barriers to work-life balance, addressing gender and maternal bias, and promoting women physician representation in leadership.

## Introduction

Physician burnout has many serious and detrimental short-term and long-term consequences, including compromised patient safety, decreased quality of care provided, increased medical errors, decreased productivity, and poor personal health [1]. It is important to address this issue early to prevent complications.

Physician burnout has been studied extensively during the last decade. In 2014, a national study in the US reported an overall physician burnout rate of 54% [2]. This is concerning, not only in and of itself, but also because this rate was nearly 10% higher than that reported among physicians just three years earlier and was approximately double that of the general population (28%) in 2014 [2]. While the situation improved slightly in 2017 with a physician burnout rate of 44%, a 2020 report documented a burnout rate among women physicians of 48%, a rate considerably higher than the burnout rate among male physicians of 37% [3]. Suicide is both more common and more deadly among physicians, with a standardized mortality ratio of 1.44 compared to the general population, with women physicians being particularly at risk [4]. Among female physicians, death by suicide is 2.27 times more likely than among non-physician women; for male physicians, death by suicide is 1.41 times more likely than among non-physician males [5]. The suicide mortality ratio (SMR) compared to non-physician individuals has been shown to be higher as well, with a SMR of 1.46 in female physicians compared to women in general and a SMR of 0.67 in male physicians compared to men in general [6].

Among physicians, burnout incidence is more influenced by external, systems-level factors than by individual-level attributes [7]. These factors adversely affect women physicians more than their male counterparts, particularly in the context of five areas: work-life integration, gender-based bias and discrimination, sexual harassment, limits to autonomy, and general workload [8].

As the COVID-19 crisis continues to engulf the world, many of the factors triggering burnout have been exacerbated. Data compiled from the beginning of the COVID-19 pandemic is already making that clear—the 2021 Medscape National Physician Burnout and Suicide Report revealed a 51% rate of burnout for women physicians versus a 36% rate for men. There are many factors that play into this inequity and these factors will likely persist as the pandemic continues to impact the world. Here, we consider how changes imposed by and during the COVID-19 pandemic are particularly challenging for women physicians, who are especially vulnerable. In particular, we discuss risk factors including response to stress, social isolation, difficulty with work-life integration, and lack of autonomy, and how these factors could be making women physicians especially susceptible to the challenges of the COVID-19 pandemic. After describing these problems, we discuss potential solutions to help mitigate the effects of the pandemic and to improve healthcare workplace environments for women physicians in the long-term.

## Discussion

### Response to stress

This pandemic has no precedent in modern times, and our best analogies may come from public health disasters that subject large populations to an external threat. The direct emotional toll of the pandemic is extremely high, similar to that of a major disaster, ranging from fear of getting infected with COVID-19 and how that poses a risk to self and loved ones to making decisions under uncertainty in both medical and professional capacities. Disaster literature reports that females have a higher risk of developing PTSD and depression compared to males [9–15], and thus we expect higher rates of PTSD and depression among women physicians as a consequence of the COVID-19 pandemic. Among these concerns, the stress of seeing family and friends affected by adverse consequences of this infection was largely a matter left to the individuals themselves, and there is no doubt such emotional stresses may impact everyone in new ways. Survey results have already demonstrated that during the COVID-19 pandemic, women are more likely to report increased worry about their job security, finances, personal health, partner’s health, and children’s health [16].

Furthermore, oncology literature shows us that female oncologists show significantly higher responses of grief, emotional stress, and burnout as a result of experiencing patient losses than do male oncologists [17]. The second victim phenomenon, which refers to the emotional distress suffered by healthcare providers after adverse events and medical errors, is also both more frequent and more intense among women physicians compared to their male counterparts [18–20]. During the early stages of the pandemic, doctors experienced unprecedented rates of patient deaths within a short time frame and may not have had the opportunity to discuss dying patients with loved ones or to debrief with colleagues. This aspect of the pandemic is also expected to have affected women physicians to a greater extent than male physicians. Further compounding the problem is that physicians, and especially female physician-parents, are often unlikely to seek help with mental issues due to concerns regarding stigma and potential impacts on career [21–26].

### Social isolation

For front-line physicians taking care of COVID-19 patients, their work is physically exhausting and time-intense, including the need for donning personal protective equipment (PPE) and proper decontamination after shifts. Compounding such time-induced social isolation, there has been the added necessity for some physicians to isolate from their family in order to protect vulnerable members. These circumstances will have compounded the social isolation mandated by the general public health measures aimed at slowing the spread of the virus. We expect that the social isolation endured by physicians during the pandemic will have affected the psychological health of women and men differently, because of the gender difference in perceived social isolation. In females, baseline social isolation and loneliness predict depressive symptoms, and these are more evident than in males [27]. During the COVID-19 pandemic, social isolation became a rule, so its impact may be unavoidable [28]. Thus, women physicians may be more vulnerable than their male counterparts when facing the unfamiliar adversary of COVID-19 alone.

### Work-life integration

Women physicians must accommodate more complexity in their work-life integration, as exemplified by the 8.5 additional hours per week on childcare and other domestic activities expended by women physicians when compared to their male colleagues [29]. The COVID-19 pandemic has created disarray in many home environments, and one might expect this to be particularly extreme for physician parents. School closures and limited availability of childcare during lockdown enforced disconnection from peer family units with younger children. Additionally, the absence of familiar stress relievers, such as down-time from day-to-day activities, likely creates unpredictable outcomes for family relationship dynamics. Women physicians working as front-line providers, particularly those with young children, may have faced extraordinary challenges with caring for the basic needs of their family. These increased childcare and household responsibilities are compounded by an increase in pandemic-related responsibilities, such as longer working hours and increased workload. These challenges may have created a new level of obstacles for work-life integration.

Many women physicians working on the front line had their professional and personal lives collide. The tragic case of Lorna Breen, a female New York City emergency physician who died from suicide, unable to cope with the new demands of the pandemic, caught national media attention. This incident highlighted the dramatic reimaging, by the popular media, of healthcare workers as a heroic, underappreciated, yet selfless, group. It is uncertain whether this focus will likely remain when the full complexity of recovery from the pandemic becomes the bigger story.

A recent survey published in June 2021 revealed that women, and especially those with children, in academic medicine were more likely than men to consider both leaving or reducing employment to part-time as a result of the COVID-19 pandemic [30]. Women were also more likely to report that they were affected by COVID-19 and stressors related to work-life balance. These stressors are likely compounding a concerning trend in which nearly 40% of women physicians go part-time or leave medicine altogether within six years of the completion of residency [31]. This is the concept of the ‘leaky pipeline,’ where women leave academic medicine at higher rates than men [32]. This phenomenon is partly explained by and contributes to the ‘glass ceiling,’ where despite increased entry of women into the medical field, their advancement into positions of power is still limited [33]. In combination, the ‘leaky pipeline’ and the ‘glass ceiling’ inherently leave women in a position more vulnerable to the consequences of the COVID-19 pandemic. It is clear that the pandemic has adversely impacted the representation of women in medicine, exacerbating disparities that already existed and reversing some of the progress that has been made. These trends are likely to continue as the pandemic continues, and the long-term implications remain to be seen.

### Autonomy

Lack of autonomy and control in the workplace is a cause of burnout that is greatly affecting women physicians [3] and it may have been exacerbated during the COVID-19 pandemic. This pandemic is unique in its creation of a suboptimal work environment for front-line providers, including lack of proper personal protective equipment (PPE) and other resources to take care of patients efficiently and safely, while protecting personal health.

While scientific academic production as a whole has increased during the pandemic, as quantified by the output of scholarly papers, the gender gap in productivity has also increased, with women academics producing fewer publications and, even when they do so, being underrepresented in the last authorship position important for promotion in the academic field [34]. Women academic physicians are less likely than men to be promoted or appointed to associate professor, full professor, and department chair positions for a variety of reasons, leading them to be under-represented in leadership [35]. This lack of representation in leadership likely had additional impact during the COVID-19 pandemic, where many structural and operational changes were implemented and leadership was responsible for making numerous decisions in response to the ever-changing conditions of the pandemic. This disparity likely also affected transparency and communication as these decisions were being made, further compounding a lack of autonomy for women physicians. Many women physicians have felt excluded from leadership in decision-making throughout the pandemic as they dealt with increases in unpaid care responsibilities [36]. In the context of the pandemic, the leadership gap is likely to have worsened underlying inequities for women physicians.

### Potential interventions

It is important to recognize that successful interventions should be multidisciplinary and systemic. Interventions to address physician burnout should include individual and organizational approaches [37–39] as burnout is not an individual phenomenon, but rather a ‘shared responsibility of healthcare systems and individual physicians’ [27]. Studies have shown that amid this COVID-19 pandemic, women in health care at are at increased risk for stress, burnout, and depression, both due to individual-level and systems-level factors [40]. The individual-level factors include lack of social support, family status, and organizational factors such as access to PPE or high workload. The systems-level factors include the prevalence of COVID-19, rapidly changing public health guidelines, and lack of recognition at work.

Physician burnout interventions specific to women should address barriers to work-life satisfaction, reduction of gender and maternal bias, mentorship opportunities, and family and pregnancy leave [41]. Burnout interventions rated with the highest importance by physicians were ‘reducing inefficient work processes and non-physician clerical work’ [42]. Micropractices, taking small moments in the day to be mindful, may be helpful to prevent burnout in the short-term, but are not meant to treat burnout [43]. There is an increasing need to incorporate physician burnout interventions at multiple levels and to address specific needs of women and ethnic minority physicians, including involving their insight in the development of interventions and programs [37].

Leaders and leadership skills have important roles in burnout prevention in general. During this pandemic, it is even more important to have leaders who are thoughtful, compassionate, caring, and take an opportunity to level the playing field. Meaningful inclusion of women, whose leadership styles are different than those of men, in healthcare leadership has also been cited as a way to promote collaborative leadership and place organizational value on physician wellness [44]. These are values that are particularly crucial during the pandemic and they contribute to the resilience of teams and the organization as a whole in coping with the effects of the pandemic. Furthermore, since women physicians were more likely to decrease or leave employment and to be unable to assume leadership positions during the pandemic, it is paramount to be mindful of the representation of women physicians in leadership, particularly when institutional policy is being crafted.

Additionally, promoting a supportive culture with stronger emphasis on work-life integration is more important than ever. It is critical to recognize the role of female physicians beyond their work environment, to create policies that can accommodate their needs, including the creation of institutional spaces that allow for childcare-sharing [45]. Access to mental health services is essential, including both connecting women physicians to community resources and creating a workplace culture where mental health is prioritized and destigmatized [45,46]. Institutional support for mental health could include measures like providing structured resilience trainings and information about different modalities of psychological support, as well as implementing peer support programs [45]. Importantly, women physicians must feel that they can seek help for symptoms of burnout without any career repercussions.

In the stressful context of the pandemic, gender bias and discrimination may go unnoticed, and it is important to have a system in place to facilitate reporting, and more importantly, improving culture. While coping with the COVID-19 pandemic has been an extremely difficult and challenging time for the physician community in general, it may have provided an opportunity to revisit and discuss these topics with greater engagement of vulnerable groups.

## Conclusions

Although the full consequences, magnitude, and depth of the impact of this crisis on the physician community and physician well-being are yet to be seen, it is already clear that the COVID-19 pandemic has had considerable repercussions for the well-being of women physicians. Given that the pandemic is likely to become a chronic part of our lives, it is particularly crucial to not turn a blind eye to this vulnerable population. With these new challenges come new opportunities to address underlying inequity and foster the well-being of women physicians and the resilience of the medical workforce as a whole.

## Data Availability

Data sharing is not applicable to this article as no new data were created or analyzed in this study.
